# Categorization of multiple sclerosis relapse subtypes by B cell profiling in the blood

**DOI:** 10.1186/s40478-014-0138-2

**Published:** 2014-09-16

**Authors:** Christopher Hohmann, Bianca Milles, Michael Schinke, Michael Schroeter, Jochen Ulzheimer, Peter Kraft, Christoph Kleinschnitz, Paul V Lehmann, Stefanie Kuerten

**Affiliations:** Department of Anatomy I, University of Cologne, Joseph-Stelzmann-Str. 9, 50931 Cologne, Germany; Department of Neurology, University Hospitals of Cologne, Kerpener Str. 62, 50937 Cologne, Germany; Department of Neurology, Caritas-Krankenhaus Bad Mergentheim, Uhlandstr. 7, 97980 Bad Mergentheim, Germany; Department of Neurology, University Hospitals of Wuerzburg, Josef-Schneider-Str. 11, 97080 Wuerzburg, Germany; Institute of Clinical Epidemiology and Biometry, Comprehensive Heart Failure Center, University of Wuerzburg, Josef-Schneider-Str. 2, 97080 Wuerzburg, Germany; Department of Pathology, Case Western Reserve University, 2103 Cornell Rd., Cleveland, OH 44106 USA; Cellular Technology Limited, 20521 Chagrin Blvd, Shaker Heights, OH 44122 USA; Department of Anatomy and Cell Biology, University of Wuerzburg, Koellikerstr. 6, 97070 Wuerzburg, Germany

**Keywords:** B cells, ELISPOT, MS, Predictive value, Relapse

## Abstract

**Introduction:**

B cells are attracting increasing attention in the pathogenesis of multiple sclerosis (MS). B cell-targeted therapies with monoclonal antibodies or plasmapheresis have been shown to be successful in a subset of patients. Here, patients with either relapsing-remitting (n = 24) or secondary progressive (n = 6) MS presenting with an acute clinical relapse were screened for their B cell reactivity to brain antigens and were re-tested three to nine months later. Enzyme-linked immunospot technique (ELISPOT) was used to identify brain-reactive B cells in peripheral blood mononuclear cells (PBMC) directly *ex vivo* and after 96 h of polyclonal stimulation. Clinical severity of symptoms was determined using the Expanded Disability Status Scale (EDSS).

**Results:**

Nine patients displayed B cells in the blood producing brain-specific antibodies directly *ex vivo*. Six patients were classified as B cell positive donors only after polyclonal B cell stimulation. In 15 patients a B cell response to brain antigens was absent. Based on the autoreactive B cell response we categorized MS relapses into three different patterns. Patients who displayed brain-reactive B cell responses both directly *ex vivo* and after polyclonal stimulation (pattern I) were significantly younger than patients in whom only memory B cell responses were detectable or entirely absent (patterns II and III; p = 0.003). In one patient a conversion to a positive B cell response as measured directly *ex vivo* and subsequently also after polyclonal stimulation was associated with the development of a clinical relapse. The evaluation of the predictive value of a brain antigen-specific B cell response showed that seven of eight patients (87.5%) with a pattern I response encountered a clinical relapse during the observation period of 10 months, compared to two of five patients (40%) with a pattern II and three of 14 patients (21.4%) with a pattern III response (p = 0.0005; hazard ratio 6.08 (95% confidence interval 1.87-19.77).

**Conclusions:**

Our data indicate actively ongoing B cell-mediated immunity against brain antigens in a subset of MS patients that may be causative of clinical relapses and provide new diagnostic and therapeutic options for a subset of patients.

## Introduction

Multiple Sclerosis (MS) is one of the most frequent neurological disorders causing disability in young adults and affects approximately 2.5 million people worldwide [1,2]. An interplay between both susceptibility genes and still unknown environmental factors is considered to be causative of the disease. Due to acute inflammatory demyelination and axonal loss with partly structural repair and recovery of function, most patients suffer from a relapsing-remitting course.

In order to develop new therapeutic strategies and a better understanding of this autoimmune disorder of the central nervous system (CNS), intensive research efforts dealing with the underlying disease pathomechanisms have been undertaken. For a long time, mainly T cells were considered as the initiator and perpetuator of the disease. However, during the last two decades, the important role of B cells as antigen presenting cells and producers of autoantibodies in the pathogenesis of MS has increasingly been appreciated [3-5]. In particular, the role of B cells in MS is hardly understood. This is surprising because intrathecal antibody synthesis and oligoclonal IgG of yet unknown specificity are a diagnostic hallmark of MS [6]. Clonally expanded B cells persist in the CNS of MS patients [7] and antibody deposition with concomitant complement activation represents the most frequently observed pattern of demyelination in MS brain lesions [8]. Consistently, plasmapheresis can be beneficial in exacerbations in relapsing forms of MS [9]. Although for decades no MS-specific autoantibody has been identified, the discovery of antibodies against the potassium channel KIR4.1 in a substantial proportion of MS patients has revived interest in antibody-mediated autoimmunity in MS [10]. Work performed in MS-like preclinical models suggests a role for B cells in initiating inflammatory responses in the CNS [11] and treatment of relapsing-remitting MS (RRMS) patients with the B cell depleting monoclonal antibody rituximab rapidly and markedly reduced active CNS inflammation [12]. A similar effectiveness was shown for alemtuzumab [13] and ofatumumab [14]. Nonetheless, today there is still no first-line treatment option in MS that specifically targets B cells and B cell subsets.

The multiple lines of evidence for a contribution of B cells to the disease pathogenesis raise the question whether a sub-typing of patients according to their B cell response in the peripheral blood is not only possible, but may also permit the identification of B cell-dependent MS, thus paving the way for a target-oriented and individualized therapy.

To this end, we have recently introduced an assay based upon the enzyme-linked immunospot technique (ELISPOT) for the detection of CNS antigen-specific B cells in the blood of patients with MS. These B cells only occurred in MS patients and were absent in healthy donors and in patients with other inflammatory and non-inflammatory neurological diseases as well as other autoimmune disorders [15]. Our previous analyses focused on measurements of the brain antigen-specific B cell response after 96 h of polyclonal stimulation. Here we extend our findings by introducing a direct *ex vivo* assay for patients with clinical manifestations of an acute MS relapse. This assay allowed us to visualize acute ongoing B cell immune responses to antigens prominent in the CNS in a subgroup of patients and to correlate this response to clinical relapse parameters.

After binding of a specific antigen to the B cell receptor and its presentation to a corresponding effector T cell, B cell proliferation and differentiation into plasma cell precursors and memory B cells occur. Whereas antibody producing plasma cells are predominantly located in the bone marrow after emigration from the lymphatic follicles, resting B lymphocytes recirculate in the body and can be converted into antibody-producing plasma cells with the help of polyclonal stimulation *in vitro*. Only in the context of a relapse and at the stage of the emigration from the lymphatic follicles to the bone marrow, plasma cells become detectable in the blood and can be directly analyzed for CNS specificity.

## Material and methods

### Patients

Thirty patients that were diagnosed with MS according to the 2005 or 2010 McDonald criteria [16], respectively, and undergoing an acute MS relapse were included in the study. Aggravation of persistent disabilities or new clinical symptoms were present for at least 24 h. Exclusion criteria comprised severe accompanying systemic or psychiatric disorders as well as a history of other autoimmune diseases. Subjects who had undergone plasmapheresis or received anti-B cell therapy were also excluded. The cohort of patients analyzed in this study contained both the RRMS (n = 24) and the secondary progressive (SPMS) (n = 6) subtype of MS. Details on all patients are provided in Table 1. The research protocol was approved by the institutional ethics committees of the Universities of Cologne and Wuerzburg. For the evaluation of disease severity the *Expanded Disability Status Scale* (EDSS) was used [17]. Additionally, we employed the tool *MS Curves*, which is based on the international *MSBase* Registry and allows the assessment of the individual disease severity [18]. Results are presented as percentiles and evaluated by means of EDSS and time since disease onset in comparison to a large cohort of patients with the same disease duration.

**Table 1:** **Demographic and disease characteristics of the patient cohort**

**Pattern**	**Patient no.**	**RRMS/SPMS**	**Sex (f/m)**	**Age at time of relapse (yrs)**	**Age at time of diagnosis (yrs)**	**Relapse EDSS** ^*^	**Treatment at time of relapse**	**Remission EDSS** ^*^	**Treatment at time of remission**	**MS severity (percentile)** ^**‡**^
**I**	1	RRMS	f	19	17	3.0	GA	2.0	GA	71
**I**	2	RRMS	f	30	15	4.0	IFN	3.0	IFN	51
**I**	3	RRMS	f	18	17	4.0	None	4.0	None	91
**I**	4	RRMS	f	42	27	6.0	GA	4.0	GA	66
**I**	5	RRMS	f	32	32	3.0	None	2.5	None	81
**I**	6	RRMS	f	29	29	4.5	IFN	4.0	IFN	91
**I**	7	RRMS	f	24	24	2.0	None	1.5	None	51
**I**	8	RRMS	f	32	32	4.0	None	2.0	None	72
**I**	9	SPMS	f	25	20	4.0	FM	4.5	None	91
**II**	10	RRMS	f	22	21	2.0	IFN	2.0	NA	73
**II**	11	RRMS	f	47	47	3.5	None	2.0	IFN	72
**II**	12	RRMS	f	34	31	4.0	None	3.0	IFN	79
**II**	13^§^	RRMS	f	59	40	6.0	None	/	/	/
**II**	14	SPMS	m	36	32	6.0	Mitox	5.0	Mitox	92
**II**	15	SPMS	f	53	50	5.5	None	5.5	None	93
**III**	16	RRMS	f	32	32	2.0	None	2.0	IFN	73
**III**	17	RRMS	f	49	38	6.0	None	6.0	FM	91
**III**	18	SPMS	m	52	37	5.5	None	5.5	None	80
**III**	19^§^	RRMS	f	38	23	6.5	None	/	/	/
**III**	20	RRMS	f	48	47	4.0	IFN	3.5	IFN	91
**III**	21	SPMS	m	52	41	2.5	GA	2.5	FM	51
**III**	22	SPMS	f	54	35	7.5	None	7.0	None	92
**III**	23	RRMS	m	40	40	2.0	None	2.0	IFN	72
**III**	24	RRMS	m	61	61	2.0	None	4.0	IFN	91
**III**	25	RRMS	f	31	30	2.0	IFN	1.0	IFN	38
**III**	26	RRMS	f	21	21	3.5	None	1.0	IFN	37
**III**	27	RRMS	f	18	18	6.0	None	1.5	None	51
**III**	28	RRMS	f	45	45	2.0	None	2.0	None	72
**III**	29	RRMS	m	52	27	4.5	None	4.5	None	66
**III**	30	RRMS	f	43	43	6.0	FA	6.0	FA	94

EDSS = Expanded Disability Status Scale; FA = fumaric acid; FM = fingolimod; GA = glatiramer acetate; IFN = interferon-β; Mitox = mitoxantrone; NA = natalizumab; RRMS = relapsing-remitting MS; SPMS = secondary progressive MS.

^*^Scores on the EDSS range from 0 to 10, with higher scores indicating a greater degree of disability.

^‡^MS severity refers to the percentile rank of each individual study patient compared to a matched MS cohort of the *MSBase* Registry. Values were determined using *MS Curves* [18].

^§^These patients were lost to follow-up.

Twenty-two patients had other neurological or other inflammatory neurological diseases (OND/OIND) including one patient with global amnesia, one patient with a psychogenic gait disorder, three patients with headaches, one patient with myopathy, one patient with myasthenia gravis, one patient with epilepsia, three patients with Parkinson’s disease, one patient with polyneuropathy, one patient with Guillain-Barré syndrome, one patient with stroke, one patient with subarachnoid hemorrhage, one patient with amyotrophic lateral sclerosis, one patient with neuroborreliosis, one patient with Ménière’s disease, one patient with vestibular neuritis, one patient with somatoform pain disorder and two patients with nystagmus.

All patients gave written informed consent and were recruited from a routine clinical care unit at the Departments of Neurology, University Hospitals of Cologne and Wuerzburg and the Caritas-Krankenhaus Bad Mergentheim. Serum samples from healthy donors were obtained from Cellular Technology Limited (Shaker Heights, OH). Peripheral blood mononuclear cells (PBMC) from healthy donors were obtained from volunteers at the participating institutions after written informed consent.

### Enzyme-linked immunospot technique (ELISPOT)

PVDF membrane 96-well ELISPOT plates (Merck Millipore, Darmstadt, Germany) were coated overnight with fresh frozen whole normal human brain lysate (30 μg/ml; Novus Biologicals, Littleton, CO), dissolved in sterile phosphate-buffered saline (PBS). We deliberately chose whole brain lysate as antigenic target taking into account that each individual patient recognizes a multitude of different tissue antigens. We suggest that the use of single antigens would have been counterintuitive also following the epitope spreading hypothesis of MS. Therefore, and particularly from a clinical point of view, the approach presented here should be the most feasible. Coating with 10% fetal bovine serum (FBS; Biochrom, Berlin, Germany) in sterile PBS served as negative control, respectively. The ELISPOT findings were controlled for the quantitative frequency of B cells in each sample by including measurements for total IgG in each donor. To this end, plates were coated with anti-human Igκ (SouthernBiotech, Birmingham, AL) at 10 μg/ml. Both whole normal human brain lysate and anti-human Igκ were titrated to their optimal concentration for use in B cell ELISPOT assays. After PBMC isolation from the blood by Ficoll-Paque (GE Healthcare Europe GmbH, Freiburg, Germany) density gradient centrifugation, PBMC were diluted in complete RPMI medium consisting of RPMI-1640 (Lonza, Cologne, Germany) and 10% FBS, 1% L-glutamine (Sigma, Schnelldorf, Germany) and 1% penicillin/streptomycin (Sigma) to a concentration of 3 × 10^6^ cells/ml. Plates were blocked with 10% FBS in sterile PBS for 2 h at room temperature. For direct *ex vivo* testing 3 × 10^5^ PBMC were plated per well and afterwards incubated for 24 h at 37°C and 7% CO_2_. In order to stimulate B cells polyclonally, PBMC were cultured at a concentration of 3 × 10^6^ cells/ml for 96 h in complete RPMI-1640 medium that contained β-mercaptoethanol (Sigma), the toll-like receptor 7/8 agonist R-848 (Enzo Life Sciences, Inc., Farmingdale, NY) and IL-2 (Peprotech, Hamburg, Germany). For testing of polyclonally stimulated B cells one million cells were plated per well and incubated for 26 h at 37°C and 7% CO_2_. Biotinylated anti-human IgG (Hybridoma Reagent Laboratory, Baltimore, MD) diluted in 1% bovine serum albumin (BSA) solution was used as a detection antibody at 0.2 μg/ml. For the direct *ex vivo* testing biotinylated anti-human IgM (Hybridoma Reagent Laboratory) at a concentration of 0.05 μg/ml was additionally used. All plates were developed with Vector Blue substrate (Vector Laboratories, Burlingame, CA) after incubation with streptavidin-alkaline phosphatase (AP) (Dako, Glostrup, Denmark) at 1:1000 dilution. Spots were analyzed on an ImmunoSpot® Series 6 Analyzer (Cellular Technology Limited).

### Enzyme-linked immunosorbent assays (ELISA)

ELISA plates (Thermo Scientific, Schwerte, Germany) were coated overnight with whole normal human brain lysate (Novus Biologicals; 10 μg/ml) or anti-human Igκ (SouthernBiotech; 2.5 μg/ml), respectively, both diluted in PBS or with PBS alone. As for the ELISPOT assay, whole normal human brain lysate and anti-human Igκ were titrated to their optimal concentration for use in the antibody ELISA. Plates were blocked with 10% FBS in PBS containing 0.05% Tween 20 for 2 h at room temperature. The plates were incubated overnight with serum at 4°C. All serum samples were diluted 1:400 in 10% FBS solution containing 0.05% Tween 20 detergent. Biotinylated anti-human IgG (Hybridoma Reagent Laboratory) diluted in 0.5% FBS/0.05% Tween 20 solution was used as a detection antibody at 0.05 μg/ml. All plates were developed with tetramethylbenzidine substrate (eBioscience, Frankurt, Germany) after incubation with streptavidin-horseradish peroxidase (eBioscience) at 1:1000 dilution. The reaction was stopped with 0.16 M sulphuric acid and the optical density (OD) in the wells was read at 450 nm using a Perkin Elmer Victor 3 1420 Multilabel Counter and Wallac 1420 software version 3.00 revision 5.

### Statistical analysis

The cut-off value for a positive B cell response measured directly *ex vivo* was determined in a cohort of n = 17 healthy donors and was set to > 1.6 spots (mean value + 3 standard deviations). Twelve of the healthy donors were retested on the consecutive day with similar results to account for day-to-day variation. The cut-off value for a brain antigen-specific B cell response after polyclonal stimulation was set to > 4.5 spots as previously established [15]. Serum samples were considered positive when the OD was at least five standard deviations above the mean value of a cohort of 69 healthy control donors. The characteristics of the patients and their disease were compared among the groups according to the B cell response status with the use of the Wilcoxon rank-sum test, which was also used to determine differences in spot size morphology. The cumulative risk of the development of a MS relapse was calculated for each group according to the Kaplan–Meier method, and differences between the groups were evaluated in a univariate analysis with the log-rank test. The Cox proportional-hazards model was used to assess the predictive value of a positive brain-specific B cell response. The relative risk of the development of a MS relapse is expressed as a hazard ratio and 95% confidence interval. P-values of less than 0.05 were considered to indicate statistical significance.

## Results

PBMC from 30 patients with MS experiencing an acute relapse were analyzed for their response to brain antigen using the ELISPOT technique. In order to differentiate between an acute and a memory B cell response, we introduced two different assay types. In a direct *ex vivo* approach PBMC were incubated on the plates for 24 h immediately after separation from the blood sample without any prestimulation. Alternatively, PBMC were tested after 96 h of polyclonal stimulation with R-848, IL-2 and β-mercaptoethanol, which is an established method for the activation of resting memory B cells [19]. Secreted antibodies were captured on the ELISPOT plates and visualized as spots that corresponded to the numbers of brain antigen-specific B cells.

### Distinct size morphology of B cell spots produced directly *ex vivo* and after polyclonal stimulation in ELISPOT assays

**Figure 1:**
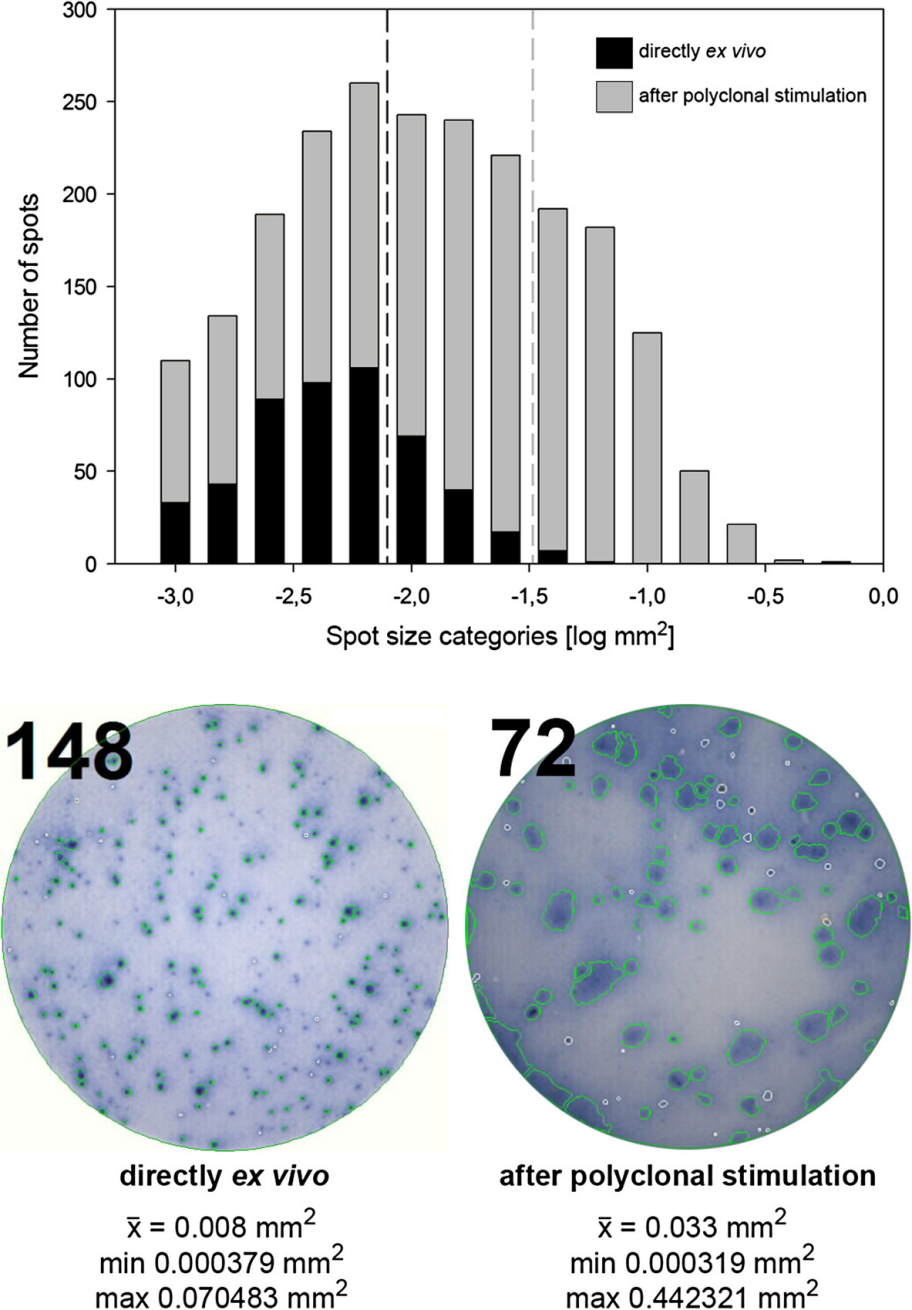
**Morphology of B cell spots measured directly** ***ex vivo*** **and after polyclonal stimulation.** The histogram shows the differences in the distribution of B cell spots measured directly *ex vivo* (black bars) or after 96 h of polyclonal stimulation (colored bars) in ELISPOT assays. The dashed lines indicate the spot size means in the two groups. A total of 524 spots were analyzed directly *ex vivo* compared to 2307 spots after polyclonal stimulation using the ImmunoSpot® software version 5.1.36 Professional DC. The images show representative wells for assays performed directly *ex vivo* or after polyclonal stimulation. The numbers in the left upper corner indicate the spot counts for the two individual wells. The mean spot size measured directly *ex vivo* was 0.008 mm^2^ compared to 0.033 mm^2^ after polyclonal stimulation. Minimum spot sizes were comparable. The maximum spot size was 0.070483 mm^2^ in direct *ex vivo* assays compared to a size of 0.442321 mm^2^ measured after polyclonal stimulation.

In a total of nine patients brain antigen-specific B cells could be detected in the blood directly *ex vivo* and after polyclonal stimulation (Table 2). We compared the morphology of spots produced directly *ex vivo* and after polyclonal stimulation using a specialized ImmunoSpot® image analysis software. Spots that were produced directly *ex vivo* were much more distinct and significantly smaller than spots produced after 96 h of polyclonal stimulation (compare a mean spot size of 0.008282 mm^2^ ± 0.00852 mm^2^ to 0.033343 mm^2^ ± 0.052619 mm^2^; p < 0.001) (Figure 1). These results underline the notion that spots detected directly *ex vivo* correspond to antibodies produced by recently activated and still recirculating plasma cells, while spots produced after polyclonal stimulation are derived from resting memory B cells that produce significantly larger spots following *in vitro* reactivation.

**Table 2:** **Distribution of B cell response patterns in patients experiencing an acute MS relapse** ^*^

**Variable**	**Pattern I**	**Pattern II**	**Pattern III**
No. of patients	9	6	15
Age – yrs			
Mean	27.9 ± 7.4	41.8 ± 13.7	42.4 ± 12.4
Range	18-42	22-59	18-61
Female sex – no. (%)	9 (100)	5 (83.3)	10 (66.7)
EDSS in remission^†^			
Mean	3.1 ± 1.1	3.5 ± 1.7^‡^	3.5 ± 2.0^‡^
Range	1.5-5.0	2.0-5.5	1.0-7.0
MS severity^¶^			
Mean	73.9 ± 16.0	81.8 ± 10.1^‡^	71.4 ± 20.2^‡^
Range	51-91	72-93	37-94
RRMS/SPMS	8/1	4/2	12/3

EDSS = Expanded Disability Status Scale; RRMS = relapsing-remitting MS; SPMS = secondary progressive MS; SD = standard deviation.

^*^Plus-minus values are means ± SD.

^†^Scores on the EDSS range from 0 to 10, with higher scores indicating a greater degree of disability.

^‡^One patient was lost to follow-up.

^¶^MS severity refers to the percentile rank of each individual study patient compared to a matched MS cohort of the *MSBase* Registry. Values were determined using *MS Curves* [18].

### Brain-reactive antibodies are detectable in the serum of patients experiencing an acute clinical relapse of MS and displaying a positive B cell ELISPOT response directly *ex vivo*

A positive response in directly *ex vivo* performed B cell ELISPOT assays should correspond to *in vivo* ongoing B cell (re)activation characterized by the secretion of antibodies by plasma cells. To confirm this assumption, we additionally obtained serum samples from n = 12 patients of our cohort during relapse and performed ELISA analysis for the detection of brain-reactive antibodies. Results are shown in Table 3. Of the 12 patients, four were characterized by a positive response in directly *ex vivo* performed B cell ELISPOT assays. These patients also displayed CNS antigen-specific antibodies in the serum as measured by ELISA. All of the eight patients that were tested negative in the directly ex vivo performed ELISPOT were also tested negative in the ELISA.

**Table 3:** **Measurements of brain-specific serum antibodies by ELISA**

**Patient no.**	**Direct** ***ex vivo*** **ELISPOT response [spot number]**	**OD**	**SD**	**OD**	**SD**	**OD brain antigen/OD total IgG**
		Brain antigen*	**Brain antigen**	**Total IgG**	**Total IgG**	
**1**	26.0 ± 4.2	1.593	0.136	2.748	0.04	0.58
**2**	9.0 ± 6.4	0.974	0.094	2.609	0.084	0.373
**3**	86.5 ± 41.7	0.852	0.107	2.693	0.049	0.316
**4**	13.0 ± 2.8	1.231	0.084	2.558	0.132	0.48
**10**	0.0 ± 0.0	0.468	0.014	2.92	0.086	0.16
**11**	0.0 ± 0.0	0.437	0.034	2.636	0.024	0.166
**12**	0.0 ± 0.0	0.108	0.000	2.673	0.024	0.04
**16**	0.0 ± 0.0	0.235	0.057	2.608	0.042	0.09
**17**	0.0 ± 0.0	0.576	0.013	2.724	0.032	0.211
**18**	0.0 ± 0.0	0.147	0.034	2.837	0.112	0.052
**20**	0.0 ± 0.0	0.369	0.047	2.729	0.12	0.135
**22**	0.0 ± 0.0	0.658	0.060	2.783	0.033	0.236

ELISA = enzyme-linked immunosorbent assay; ELISPOT = enzyme-linked immunospot technique; OD = optical density; SD = standard deviation.

*All samples were tested in duplicate wells and are represented as mean medium-subtracted values. The cut-off value for a positive response was calculated from the means of a group of 69 healthy control donors + 5 SD (OD brain antigen/OD total IgG > 0.307).

### Ongoing brain-specific B cell activity is detectable in the blood in a subset of patients with an acute disease relapse

**Figure 2:**
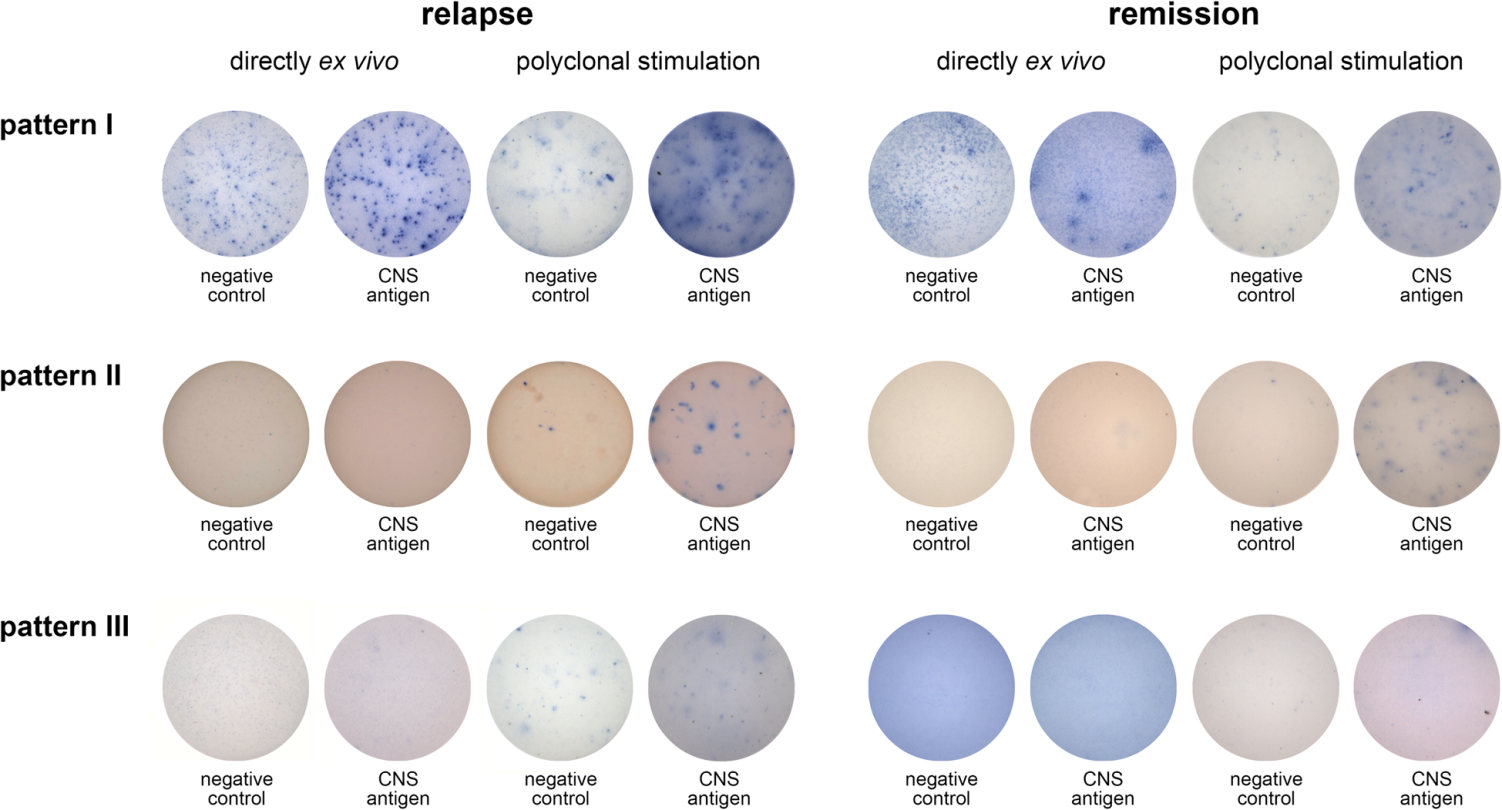
**Representative ELISPOT wells of the three different CNS antigen-specific B cell response patterns.** Pattern I: positive brain antigen-specific B cell response directly *ex vivo* and after polyclonal stimulation during relapse, persisting memory B cell response in remission. Pattern II: positive brain antigen-specific B cell response only after polyclonal stimulation both in relapse and remission. Pattern III: absence of brain antigen-specific B cell responses at any time point. Images and pattern classifications are representative for a study cohort of 30 patients.

In addition to the nine patients that showed a positive B cell response both directly *ex vivo* and after polyclonal stimulation, we identified six of 30 patients that did not show any B cell activity associated with the relapse, but were classified as B cell positive donors only after polyclonal stimulation. The remaining 15 of 30 patients displayed brain antigen-specific B cells neither in direct *ex vivo* assays nor after 96 h of stimulation. These data suggest that the MS patients tested in our study fall into three different categories, depending on the presence of an actively ongoing and/or CNS antigen-specific memory B cell response in the blood. These categories are summarized as “patterns” in Tables 1 and 2 and Figure 2. No significant differences were found between the patterns in relation to relapse or disease severity (Table 2). However, patients who were classified as “pattern I” with a positive B cell response both directly *ex vivo* and after polyclonal stimulation were significantly younger than the cohort of patients represented by both pattern II and III (p = 0.003). In order to delineate that the CNS antigen-specific B cell response was not a transient phenomenon, but a characteristic feature of a disease subtype, we retested 21 MS patients in clinical remission three to nine months after relapse. In 17 of 21 patients the CNS antigen-specific B cell response detected after polyclonal stimulation was comparable to the results obtained during relapse. Four patients who had been tested negative initially, now showed a positive response. Loss of a brain antigen-specific B cell response in previously positive patients was not observed. Importantly, the formerly evident B cell activity in nine of 30 patients as measured directly *ex vivo* was absent in remission. Brain antigen-specific B cells were also absent directly *ex vivo* in healthy donors (n = 17) and patients with other neurological or other inflammatory neurological diseases (n = 22).

### Association between the development of brain-specific B cell responses in the blood and acute disease reactivation

**Figure 3:**
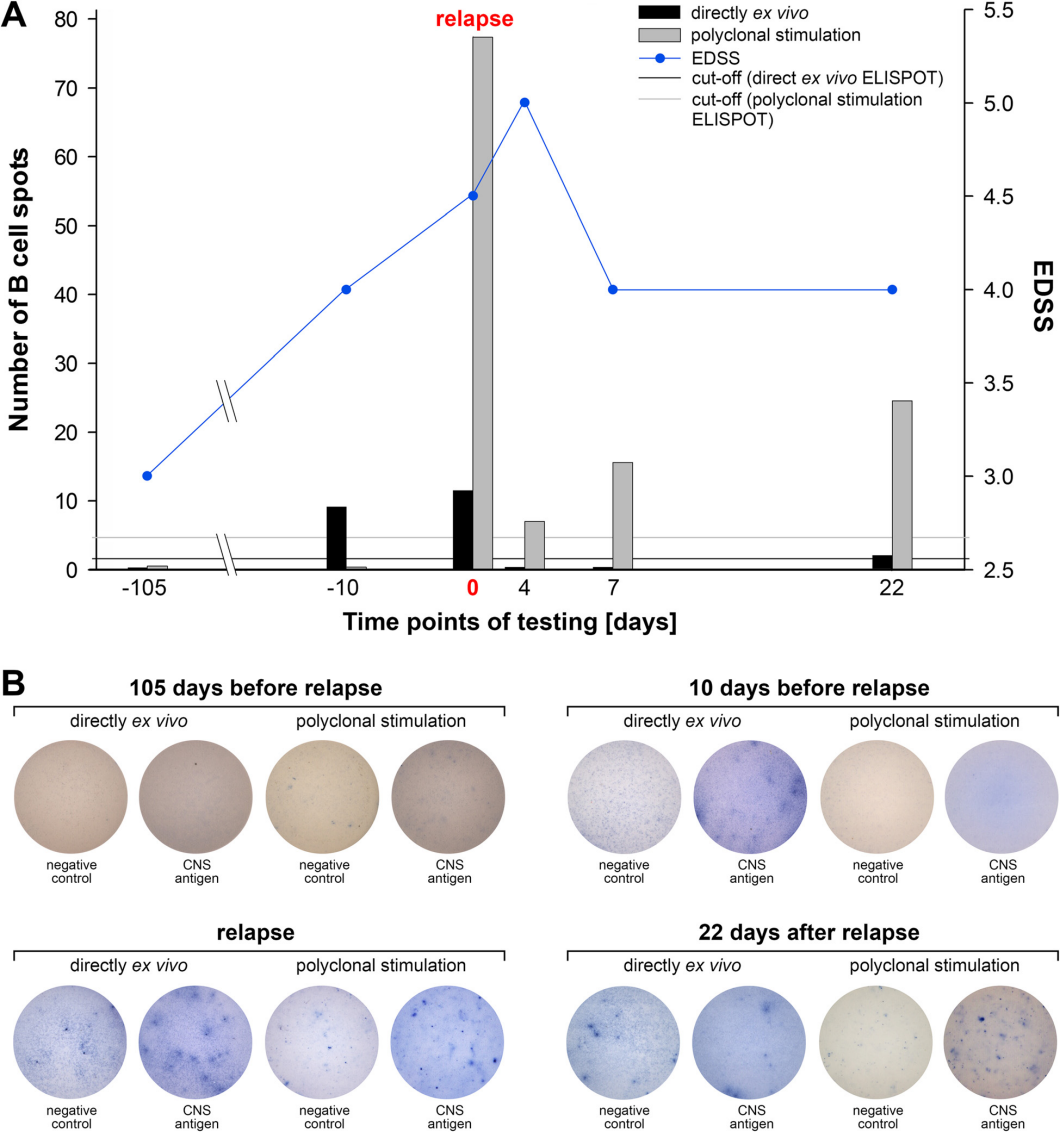
**ELISPOT results of a patient showing a conversion from pattern III to pattern I.** Panel **A** shows the number of B cell spots measured in ELISPOT assays directly *ex vivo* and after 96 h of polyclonal stimulation in an individual patient on six consecutive time points. The severity of clinical disease is represented by the EDSS score that is referred to by the line graph. Representative well images of four different time points are shown in Panel **B** depicting the development of a brain antigen-specific B cell response in the course of the disease.

One interesting finding pertained to a patient who originally did not display any CNS antigen-specific B cell responses directly *ex vivo* and after polyclonal stimulation. In this patient clinical examination three months after relapse revealed a recovery of function and no signs for a renewed clinical disease exacerbation despite the persistence of paresthesia. However, a positive brain antigen-specific B cell response was evident in direct *ex vivo* ELISPOT testing indicating recent immune reactivation. Ten days later the patient was admitted to the hospital with symptoms of a clinical relapse including deficits in visual, cerebellar, sensory as well as motor functions. ELISPOT analysis confirmed the presence of plasma cells actively secreting brain antigen-reactive antibodies. The patient was examined at three subsequent time points during which we were able to demonstrate the disappearance of brain antigen-specific plasma cells from the blood. At the same time, a brain antigen-specific memory B cell response became detectable (Figure 3). The dramatic drop in the number of B cell spots measured directly *ex vivo* and after polyclonal stimulation within four days between the third and fourth measurement may be explained either by the high dose intravenous glucocorticoid therapy that was administered at the time point of relapse for three consecutive days [20] and/or by the migration of the autoreactive B cells into the target tissue, that is the CNS.

### A positive brain-specific B cell response in the blood is predictive of a subsequent relapse

**Figure 4:**
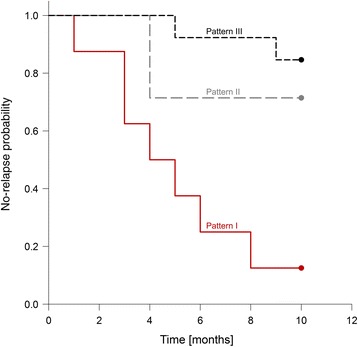
**Kaplan–Meier estimates of the risk of a MS relapse according to the presence of brain-specific B cells in the blood.** P = 0.0005 for the comparison between patients who were tested positive for direct *ex vivo* brain-specific B cells (pattern I) and patients who were tested negative (patterns II and III).

The association between a positive brain-reactive B cell response in the blood in direct *ex vivo* assays and the development of a consecutive relapse as shown for one case in Figure 3 suggests that the presence of brain-reactive B cells in the blood could be linked to a higher relapse rate. To test this hypothesis, we followed n = 8 patients that displayed a brain-reactive B cell response in the blood directly *ex vivo* and after polyclonal stimulation (pattern I), n = 5 patients that displayed brain-reactive B cells only after polyclonal stimulation (pattern II) and n = 14 patients with a negative B cell response in both assays (pattern III). The patients were followed for a period of ten months and the time to the next relapse after initial testing was recorded. Data are summarized in Table 4. The Kaplan-Meier plot shows that the relapse-free interval in patients that were tested positive in direct *ex vivo* ELISPOT assays was significantly shorter than in patients that were classified as pattern II or III (p = 0.0005) (Figure 4). There was no significant difference between patients with pattern II *versus* pattern III (p = 0.348). The hazard ratio for the development of a consecutive relapse in the setting of a positive direct *ex vivo* brain-reactive B cell response in the blood (pattern I) compared to patients with pattern II or III was 6.08 (95% confidence interval 1.87-19.77).

**Table 4:** **Numbers of patients at risk in the different B cell response groups in the Kaplan-Meier analysis**

**Time [months]**	**Pattern I**	**Pattern II**	**Pattern III**
**0**	8	5	14
**1**	7	5	14
**2**	7	5	14
**3**	5	5	14
**4**	4	3	14
**5**	3	3	12
**6**	2	3	12
**7**	2	3	12
**8**	1	3	12
**9**	1	3	11
**10**	1	3	11

## Discussion

In the pathogenesis of MS, multiple lines of evidence indicate an important role of B cells and autoantibodies [21-24]. For decades, intensive research efforts were made to identify the target antigen in MS, but antibodies against myelin components or other structures prominent in the CNS were also frequently found in other neurological diseases and/or healthy individuals [4,25]. The assumption that antibodies are pathogenic in the development of MS is mainly supported by histopathological findings that provided evidence for the frequent deposition of immunoglobulins and complement factors in MS brain lesions [8]. In the past an association between the neuropathological pattern II defined by Lucchinetti et al. and a benefit from plasma exchange was suggested [26] and treatment with the B cell-specific monoclonal antibodies rituximab, alemtuzumab or ofatumumab were effective in ameliorating disease severity by means of reduction in the number of total gadolinium-enhancing magnetic resonance imaging (MRI) lesions as well as lower annualized relapse rates [12-14]. It has initially been suggested that in particular antibodies against myelin oligodendrocyte glycoprotein (MOG) and myelin basic protein (MBP) were predictive of a conversion from a clinically-isolated syndrome (CIS) to clinically definite MS [27]. However, a subsequent corroboration of these results failed [28]. Here we show that the presence of a brain-specific memory B cell response in the blood as measured by ELISPOT was associated with an increased risk of the development of a MS relapse.

In our previous work we have demonstrated that the detection of brain antigen-specific B cells in the PBMC population permitted the identification of a B cell-dependent subtype of MS [15]. These data were in line with earlier reports that showed the presence of proteolipid protein (PLP)- and MOG-specific B cells in the blood and cerebrospinal fluid (CSF) of patient with MS using the ELISPOT approach [29,30]. The data presented here extend these findings not only by presenting the predictive value of this test, but also by the introduction of a direct *ex vivo* assay that indicated actively ongoing disease and was associated with clinical disease reactivation in a fraction of patients. It is tempting to speculate that the presence of brain-reactive B cell responses in the blood can be used to subdivide MS patients into different categories as suggested in the current study. A categorization of patients has already been done following different patterns of demyelination in brain lesions and the most frequently observed pattern was characterized by the deposition of antibodies. Interindividual heterogeneity in the patterns of demyelinative pathology has been suggested [8]. Our data support this concept, but imply that there might also be intraindividual heterogeneity in regard to the brain-specific B cell response over the course of the disease.

Acute clinical relapses of MS often lead to the deterioration of clinical symptoms and the failure of functional CNS systems. As yet, the evidence-based standard treatment for MS relapses is a high-dose intravenous glucocorticosteroid pulse therapy. Since relapse treatment can only be initiated when new clinical symptoms of MS are evident and persist, there is always a risk of the development of irreversible deficits. The ability to detect relapses before they become clinically evident and their consecutive early treatment would provide an option to prevent the accumulation of CNS damage. A subtyping of blood immune responses as suggested here might be one possible option along these lines. Even if the CSF is also relatively easy to access and it might be argued that immune responses in the CSF reflect the pathogenic processes in the CNS more closely, the risks of side effects and the ethical problems with exposing patients to repeated spinal taps emphasize the clinical and practical advantage of a test that can be performed on peripheral blood.

## Conclusion

The data presented here strengthen the central role of B cells in the immune pathogenesis of MS. It is conceivable that our results will help to identify patients with B cell-/antibody-dependent MS and relapses, thereby guiding the development and use of B cell-directed therapeutic strategies. It remains to be elucidated if the detection of recirculating B cells that produce CNS-specific antibodies *ex vivo* will allow the diagnosis of MS reactivation even before the occurrence of clinically evident symptoms, which would help to facilitate the initiation of early treatment that could potentially include plasmapheresis [26]. Finally, the detection of antibody-producing B cells in MS patients corroborates the autoimmune hypothesis of the disease and its association with clinical disease parameters.

## Abbreviations

AP: Alkaline phosphatase
BSA: Bovine serum albumin
CIS: Clinically-isolated syndrome
CNS: Central nervous system
CSF: Cerebrospinal fluid
EDSS: Expanded disability status scale
ELISA: Enzyme-linked immunosorbent assay
ELISPOT: Enzyme-linked immunospot technique
FBS: Fetal bovine serum
MBP: Myelin basic protein
MOG: Myelin oligodendrocyte glycoprotein
MRI: Magnetic resonance imaging
MS: Multiple sclerosis
OD: Optical density
OND: Other neurological diseases
OIND: Other inflammatory neurological diseases
PBMC: Peripheral blood mononuclear cells
PBS: Phosphate-buffered saline
PLP: Proteolipid protein
RRMS: Relapsing-remitting multiple sclerosis
SD: Standard deviation
SPMS: Secondary progressive multiple sclerosis
